# ASO Author Reflections: Developing the GRAFT Score for Skin Graft Failure After Skin Cancer Excision

**DOI:** 10.1245/s10434-026-19910-9

**Published:** 2026-06-02

**Authors:** Hasan Asfour, Khalid Aram, Mohamed Hassouna, Reena Agarwal

**Affiliations:** 1 Leicester Cancer Research Centre, College of Life Sciences, University of Leicester, Leicester, UK; 2 Department of Plastic Surgery, University Hospitals of Leicester NHS Trust, Leicester, UK; 3 School of Business and Technology, Emporia State University, Kansas, USA

## Past

Skin reconstruction using skin grafts is commonly performed after skin cancer resection. Despite its widespread use, graft failure remains a clinically important complication. Current literature has identified potential contributors to graft failure, but the evidence for integrated clinical prediction is lacking.^1–3^

This study therefore addressed two main clinical questions. First, which preoperative clinical variables independently can predict skin graft failure after skin cancer resection? Second, can these variables be combined into a clinically practical prediction model?

To address these questions, the authors developed and internally validated the skin Graft Risk Assessment of Failure Tool (GRAFT) score using a retrospective patient cohort undergoing skin-grafting after skin cancer resection. Their goal was to prioritize simplicity, reproducibility, and bedside applicability using predictors available before surgery.

## Present

In this study, anticoagulation and lower body location of skin cancer were the strongest independent predictors of graft failure and formed the basis of the final multivariable model. Both predictors are readily available before surgery, making the model practical for clinical use.^4^

The GRAFT score demonstrated acceptable discrimination and good calibration, with stable performance after internal validation, supporting the robustness of the model. By developing a simple points-based scoring system, the model allows clinicians to stratify patients into three risk groups (low-, intermediate-, and high-risk groups) at the bedside without further investigations or complex calculations.

Identification of high-risk patients before surgery may inform individualized perioperative strategies, including preoperative optimization, meticulous intraoperative assessment of wound bed and hemostasis, and closer postoperative follow-up evaluation. The GRAFT score also supports better informed consent by providing objective estimates of complication risk.

## Future

Although the GRAFT score demonstrated encouraging internal validity, external validation in independent multicenter cohorts is essential to evaluate the generalizability of the model. Such validation also should assess performance across varying graft types, anticoagulation regimens, and anatomic subsites.

Future works should additionally explore incorporation of other clinically relevant variables that were not consistently captured in this retrospective dataset, including other comorbidities such as hypertension, intraoperative hemostatic techniques, bolster fixation methods, postoperative immobilization adherence, and surgeon-specific practices. Prospective studies also may clarify whether dynamic perioperative variables improve predictive performance.

Importantly, prediction alone does not improve outcomes unless linked to actionable interventions. Future works should therefore investigate whether risk-guided perioperative management strategies informed by the GRAFT score can reduce graft failure rates, improve patient outcomes, and enhance health care cost-effectiveness. Ultimately, the authors envision the GRAFT score as a clinically practical framework that supports clinical judgment. With further validation and refinement, this model may help standardize risk communication and perioperative planning for patients undergoing skin graft reconstruction after skin cancer excision.
